# Supplementary material for: Ankyrin-G mediates targeting of both Na+ and KATP channels to the rat cardiac intercalated disc
Source: eLife. 2020 Jan 14;9:e52373. doi: 10.7554/eLife.52373 (PMC7299345; doi:10.7554/eLife.52373)
Supplement: Supplementary file 1. [file elife-52373-supp1.docx]

# Ankyrin-G Mediates Targeting of both Na^+^ and K_ATP_ Channels to the Cardiac Intercalated Disc

# Supplementary Tables

Table S1: Validation of antibodies

| Subunit | Ab Name | System | Technique used | Validated in this study | Validated in the literature (PMID) |
| --- | --- | --- | --- | --- | --- |
| Nav1.5 | Sigma S8809 | HEK-293 | Immunoblotting | x |  |
| Nav1.5 | Sigma S0819 | Heart | Immunostaining | x |  |
| Kir6.2 | Self-developed C62 | Heart | Immunostaining |  | 22245446 |
| Kir6.2 | Santa Cruz N18 | HEK-293 | Immunoblotting | x |  |
| Kir6.2 | Lee62 | Heart | Immunostaining | x |  |
| AnkG | Neuromab clone N106/20 | Heart | Immunostaining |  | 26024478 |
|  |  | HEK-293 | Immunoblotting |  |  |
| AnkB | Neuromab clone N105/17 | Heart | Immunostaining |  | 25362471 |
|  |  | HEK-293 | Immunoblotting |  |  |
| Myc | Sigma 9E10 | HEK-293 | Immunoblotting | x |  |
| GAPDH | Sigma G8795 | HEK-293 | Immunoblotting |  |  |
| Caveolin3 | C38320 | Heart | Immunoblotting |  | 11259414 |
